# Leiomyosarcoma in the mandible: A rare case report

**DOI:** 10.1097/MD.0000000000004011

**Published:** 2016-07-08

**Authors:** Bogumił Lewandowski, Robert Brodowski, Paweł Pakla, Wojciech Stopyra, Iwona Gawron

**Affiliations:** a Department of Maxillo-Facial Surgery, Fr. Chopin Clinical Voivodeship Hospital; b Chair of Emergency Medical Service, Faculty of Medicine, the University of Rzeszow; c Department of Radiotherapy, Subcarpathian Oncology Centre, Rzeszow, Poland.

**Keywords:** adjuvant radiotherapy, surgery, leiomyosarcoma, mandible, mouth

## Abstract

Leiomyosarcoma (LMS) is a malignancy which very rarely occurs in maxillofacial location, and the course of the disease is not very characteristic.

In this case report, we present a 58-year-old female patient with a painless tumor of the left angle of the mandible causing slight asymmetry of the face. She also reported that she observed deterioration in fitting of the lower denture in the oral cavity for several months, which she had used successfully for 5 years.

On the basis of clinical tests, histopatological examination, and imaging (CT, MRI, ultrasound, pantomography), the patient was diagnosed with primary malignant leiomyosarcoma (LMS) of the mandibular corpus and ramus on the left side. The patient received combined surgical and oncological treatment. The first stage was a surgery, and then adjuvant radiotherapy was applied on the site of the resected tumor—a total dose of 60 Gy in 35 fractions. The patient's postoperative course was uneventful. She also underwent adjuvant therapy well. In the period of 3-year follow-up, no signs of recurrence were observed.

The findings may extend our knowledge and experiences in the treatment of leiomvosarcoma in the craniofacial area.

## 1 Introduction

Sarcomas are a rare group of malignant mesenchymal tumors. They account for about 1% of all malignancies in adults. Histologically, there are 2 types of leiomyosarcomas: more frequent rhabdomyosarcoma (RMS) formed from striated muscle cells and rarely encountered leiomyosarcoma (LMS) developing of smooth muscle mainly in the gastrointestinal tract, bladder, and retroperitoneal space.^[1]^ Leiomyosarcomas constitute about 3% to 10% of all sarcomas in the head and neck area. Sarcomas of smooth muscle origin, LMS, are extremely rare in the area of the mouth and jaws. This is due to the rarity of smooth muscle in that area, that is, in the vessel walls, arrector pilli muscles, circumvallate papillae, and myoepithelial cells of salivary glands.^[2,3]^ Only 65 cases of leiomyosarcoma (LMS) located in the mandible were described in the English literature in 1950 to 2011.^[4]^

Leiomyosarcoma is a malignant tumor occurring most frequently in mature age, especially in the sixties.^[5]^ Cases in a wide range of ages (1–88 years) were also described in the specialist literature.^[6]^ This type of malignancy is slightly more common in men than women. The group of the etiological factors distinguished in the specialized literature included: predisposing disease (chronic lymphedema, diseases accompanied with immunosuppression, viral diseases), environmental factors (radiation, trauma, foreign body, chemical compounds: herbicides, pesticides), and genetic disorders (neurofibromatosis, Gardner's syndrome, Li–Fraumeni syndrome).^[7]^

LMSs grow slowly in the initial period and do not have characteristic symptoms. Leiomyosarcoma usually manifests in the mouth in the form of significantly malignant tumor. Previously described locations were related to the tongue, mandible, palate, cheeks, maxillary sinuses, upper and lower gingiva. Initially, LMS affects gingival soft tissues as a nonulcerated swelling that can occur on the buccal and lingual tissue.

This kind of tumor is characterized by rapid growth progression and requires combined treatment: radical surgery and then adjuvant radiotherapy.

Reported size of tumors ranged from 1 to 10 cm. Immunohistochemistry plays an important role in diagnosis and differentiation from other tumors with similar histopathological image. Leiomyosarcomas are composed of spindle cells with visible smooth muscle differentiation and distinct features of atypia. Immunohistochemical tests indicate the presence of actin and desmin, which are detected in 100% and 50% of cases, respectively. Sarcomas tend to spread mainly through the circulatory system (most commonly to the lungs, 10–30% of cases), and rarely through the lymphatic system (to the lymph nodes).^[8]^ Apart from histological examination, imaging examinations such as CT and MRI are used in the diagnosis of LMS in order to accurately determine the size, location of the tumor, and its relation to surrounding anatomical structures. The size and the location of the tumor are primary factors which allow us to determine prognosis. Patients with small tumors located in the area allowing for a radical resection with wide margin of healthy tissue have a greater chance of recovery. Radical tumor resection is crucial. Unfortunately, anatomical conditions in the head and neck cause an increased risk of too narrow margin of healthy tissue. The patient remains under strict oncological supervision for at least 5 years.

The aim of the study was to present a case of a patient treated at Clinical Department of Maxillo-Facial Surgery in Rzeszów due to a leiomyosarcoma in the left angle of the mandible.

## 2 Case study

The patient aged 58 years was referred to the Department of Maxillo-Facial Surgery in Rzeszów due to a painless tumor of the left angle of the mandible causing slight asymmetry of the face. On admission, the patient reported that she observed deterioration in fitting of the lower denture in the oral cavity for several months, which she had used successfully for 5 years. At the same time, the patient also noticed discomfort in the left angle of the mandible that she associated with problems in lower denture fitting, and therefore the patient visited a dentist to ask for a new prosthetic restoration. The doctor referred her to the local department of Maxillo-Facial Surgery.

**Figure 1:**
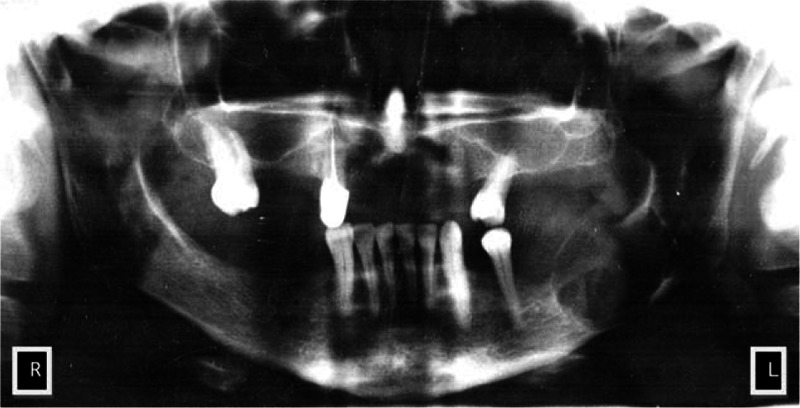
The tumor of the left mandibular angle in the pantomography. Spherical thinning of bone structure with osteosclerotic border in the left mandible angle.

On admission, soft tissue swelling causing a slight asymmetry in the left mandibular angle was found in the patient. Submandibular lymph nodes on the left were enlarged, movable, and slightly tender. Pain was observed at wide opening of the jaws. A tumor located in the retro-molar space in the mandible, mainly from the lingual side, was found in the intraoral examination with the diameter of 35 to 40 mm covered with unchanged mucosa. It was painless on palpation with clearly demarcated upper border of the tumor. A cyst was suspected in the patient on the basis of a preliminary examination. Pantomography showed spherical thinning of the bone structure with osteosclerotic border in the left mandibular angle (Fig. 1).

The patient was referred to microscopic tumor verification—excisional biopsy. The procedure was performed under general endotracheal anesthesia with intraoral access. Intraoperative examination revealed a damaged cortex of the mandible and osteolytic bone loss of 3- to 3.5-cm diameter, which was filled with a tissue mass of lard consistency easily separated from the bone foundation. The surgical wound was sutured with simple interrupted sutures. The removed tumor in 10% formalin solution was sent for histopathological examination. Microscopic preparations were consulted in the Department of Pathology, Collegium Medium, Jagiellonian University in Kraków. The test No. 1776506: leiomyosarcoma (low grade). Immunohistochemical profile (IHC): SMA + Vimentyna + Desmina-, S100-, Ki67 + 10%.

**Figure 2:**
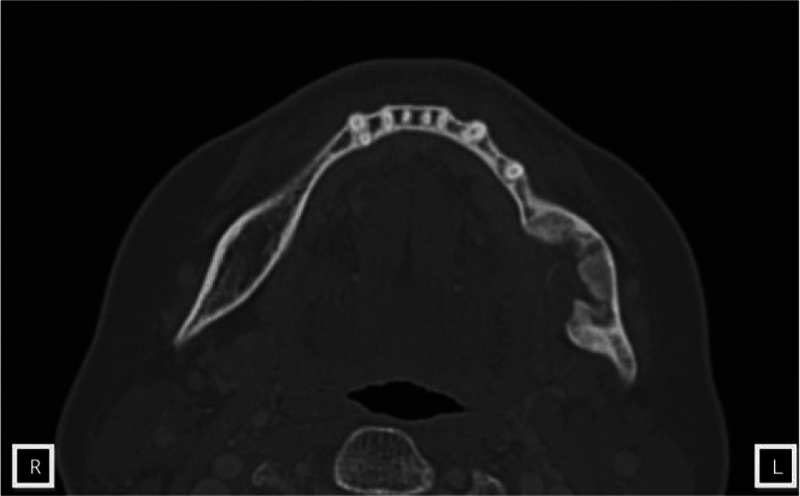
The tumor of the left mandibular angle in CT scan. Irregular loss in the bone structure sized 30 × 37 × 20 mm. CT = computed tomography.

**Figure 3:**
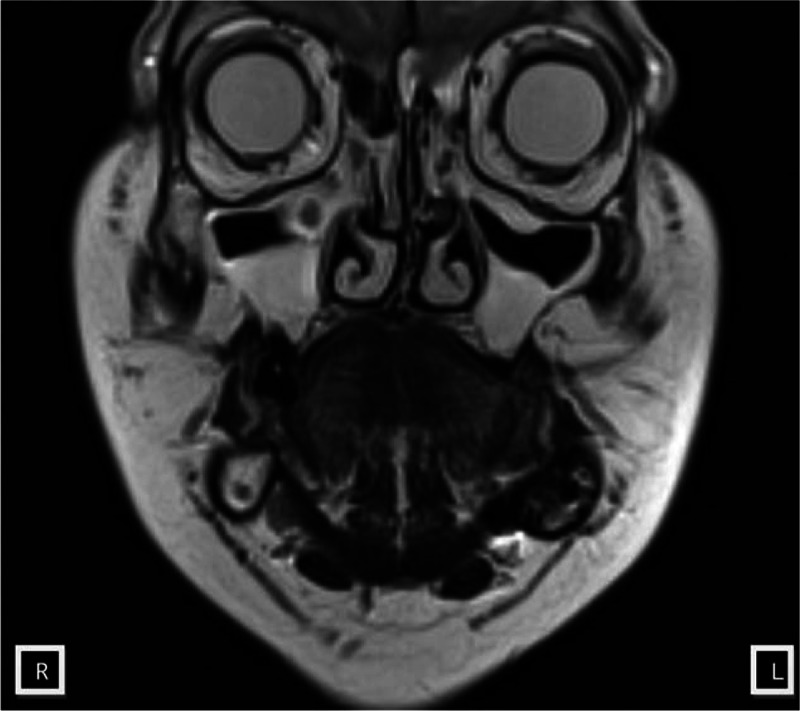
The tumor of the left mandibular angle in MRI scan. Bone loss filled with a tumor of irregular shape sized 40 × 30 × 18 mm in the left angle and mandibular ramus.

The diagnosis was extended in connection with the results obtained in the histopathological examination. CT and MRI of the head and facial skull, ultrasound of the neck and abdomen, chest X-ray were performed. CT of the head and neck showed irregular loss in the bone structure sized 30 × 37 × 20 mm; the surrounding bone structure of uniformly increased sclerosis was filled with hypodense palpable mass without contrast enhancement (Fig. 2). MRI revealed bone loss which was filled with a tumor of irregular shape sized 40 × 30 × 18 mm located in the left angle and the mandibular ramus. Cortical bone destruction was found mainly at the lingual and buccal side (Fig. 3). Ultrasound of neck lymph nodes did not show any pathological changes and a single nodular lesion was found within the right lobe of the thyroid sized 14 × 22 × 31 mm. Fine needle aspiration biopsy (BAC) revealed the presence of lesion of benign adenoma character in the thyroid gland. Abdominal ultrasound and chest x-ray showed no pathologies.

**Figure 4:**
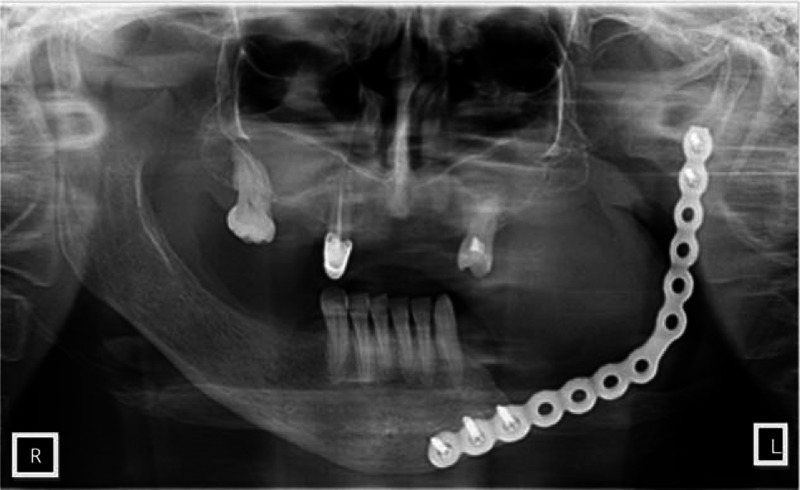
The pantomography—3 years after the partial resection of the corpus and ramus of the mandible with reconstruction of bone by means of titanium plate.

On the basis of clinical tests, histopatological examination, and imaging (CT, MRI, ultrasound), the patient was diagnosed with primary malignant leiomyosarcoma (LMS) of the mandibular corpus and ramus on the left side. The patient was referred to combined surgical and oncological treatment. The first stage was a surgery, and then adjuvant radiotherapy. The patient was qualified for extended surgery, that is, partial resection of the corpus and ramus on the left and excision of groups I, II, and III lymph nodes with simultaneous restoration of bone continuity by means of a titanium plate. The resection of mandibular corpus and ramus, and the titanium plate reconstruction were performed under general anesthesia. Immediately after surgery, the patient was monitored in the postoperative room. The patient's postoperative course was uneventful, and in the following days, her general condition was good. Granulation and prolonged wound healing were observed locally in the distal part of the wound. Redon drain was removed 5 days after the surgery. The patient reported, after the surgery, hypoesthesia of the lower lip, chin, and the mucous membrane of the mouth bottom on the left. After 10 days of surgery, the patient was discharged from the clinic and transferred to the Department of Radiotherapy, Subcarpathian Oncology Centre, where adjuvant radiotherapy was applied on the site of the resected tumor—a total dose of 60Gy in 35 fractions. From the 10th day of radiotherapy radiation, lesions were observed on the face, neck, and oral mucosa. She underwent adjuvant therapy well. The patient is under supervision of Oncology Clinic, Subcarpathian Oncology Centre, Rzeszow, until now. CT examination of facial part of cranium and neck did not reveal any local recurrence. Mandibular condyle head was movable on palpation, opening of the jaws was somewhat limited to ∼3 cm. The scar in the chin and submandibular area on the left was linear and smooth, with no signs of recurrence; the left cheek was slightly hollowed. Intraoral examination showed the presence of scar tissue on the bottom of the oral cavity on the left without characteristics of local recurrence. The surrounding soft tissues had increased density (Fig. 4). In the period of 3-year follow-up, no signs of recurrence were observed. The patient gave written informed consent for this publication.

## 3 Discussion

A very rare occurrence and lack of characteristic symptoms of leiomyosarcoma developing in maxillofacial area poses a significant diagnostic and therapeutic challenge in clinical practice.

Difficulties in diagnosis of the described case were associated with less distinctive microscopic image of collected slice and surgical material. Consultation of preparations in Reference Centre contributed to proper diagnosis and initiation of treatment. In addition to the standard histopathological examination, immunohistochemical profile: SMA + Vimentyna + Desmina-, S100-, Ki67 + 10% plays an important role in LMS diagnostics; it allows us to differentiate tumors with similar histopathological image, that is, rhabdomyosarcoma, fibrosacoma, angiosarcoma, and the others.

Only isolated cases of LMS located in the mouth were described in the Polish specialist literature. Yan et al^[9]^ conducted metanalysis of 20 LMS cases treated during 37 years in West China Stomatology Hospital. The authors reported that LMS located in the maxillo-facial area is very rare, and its incidence did not exceed 0.06% of all malignant tumors developing in the area of the oral cavity.

The presented case of 58-year-old woman did not correlate with etiologic factors.

According to modern views on LMS therapy, combined therapy is effective (surgical treatment with adjuvant radiotherapy and chemotherapy). The most important predictor is microscopic image of free surgical margins. According Ethunandan et al,^[10]^ the 5-year survival rate for primary LMS in the oral cavity is ∼55%. Local recurrence occurs in ∼34% of cases. The regional lymph nodes are affected in >15%.^[11]^ Distant metastases, mostly to the lung, occur in ∼35% of patients with LMS tumor in the oral cavity.^[12]^ The issue of the LMS treatment is a major challenge for a surgeon and an oncologist. Surgical treatment was applied in the patient treated in our clinic; it consisted of tumor excision with partial resection of the mandible and reconstruction of a tissue defect with titanium plate and adjuvant radiotherapy. The patient had uneventful perioperative course.
